# Modification of Oil Palm Mesocarp Fiber Characteristics Using Superheated Steam Treatment

**DOI:** 10.3390/molecules18089132

**Published:** 2013-07-30

**Authors:** Noor Ida Amalina Ahamad Nordin, Hidayah Ariffin, Yoshito Andou, Mohd Ali Hassan, Yoshihito Shirai, Haruo Nishida, Wan Md Zin Wan Yunus, Subbian Karuppuchamy, Nor Azowa Ibrahim

**Affiliations:** 1 Department of Bioprocess Technology, Faculty of Biotechnology and Biomolecular Sciences, Universiti Putra Malaysia, Serdang 43400 UPM, Selangor, Malaysia; E-Mails: idamalina@yahoo.com (N.I.A.A.N.); alihas@biotech.upm.edu.my (M.A.H.); 2 Faculty of Chemical and Natural Resources Engineering, Universiti Malaysia Pahang, Lebuhraya Tun Razak, Kuantan 26300, Pahang, Malaysia; 3 Laboratory of Biopolymer and Derivatives, Institute of Tropical Forestry and Forest Products (INTROP), Universiti Putra Malaysia, Serdang 43400 UPM, Selangor, Malaysia; 4 Department of Biological Functions and Engineering, Graduate School of Life Science and System Engineering, Kyushu Institute of Technology, 2-4 Hibikino, Wakamatsu, Fukuoka 808-0196, Japan; E-Mails: yando@life.kyutech.ac.jp (Y.A.); shirai@life.kyutech.ac.jp (Y.S.); nishida@lsse.kyutech.ac.jp (H.N.); 5 Faculty of Defense Science and Technology, National Defence University of Malaysia, Kuala Lumpur 57000, Malaysia; E-Mail: wanmdzin@upnm.edu.my; 6 Department of Energy Science, Alagappa University, Karaikudi, Tamilnadu, India; E-Mail: skchamy@gmail.com; 7 Department of Chemistry, Faculty of Science, Universiti Putra Malaysia, Serdang 43400 UPM, Selangor, Malaysia; E-Mail: norazowa@science.upm.edu.my

**Keywords:** oil palm mesocarp fiber (OPMF), silica bodies, superheated steam treatment (SHS), hemicelluloses removal, biocomposite

## Abstract

In this study, oil palm mesocarp fiber (OPMF) was treated with superheated steam (SHS) in order to modify its characteristics for biocomposite applications. Treatment was conducted at temperatures 190–230 °C for 1, 2 and 3 h. SHS-treated OPMF was evaluated for its chemical composition, thermal stability, morphology and crystallinity. OPMF treated at 230 °C exhibited lower hemicellulose content (9%) compared to the untreated OPMF (33%). Improved thermal stability of OPMF was found after the SHS treatment. Moreover, SEM and ICP analyses of SHS-treated OPMF showed that silica bodies were removed from OPMF after the SHS treatment. XRD results exhibited that OPMF crystallinity increased after SHS treatment, indicating tougher fiber properties. Hemicellulose removal makes the fiber surface more hydrophobic, whereby silica removal increases the surface roughness of the fiber. Overall, the results obtained herewith suggested that SHS is an effective treatment method for surface modification and subsequently improving the characteristics of the natural fiber. Most importantly, the use of novel, eco-friendly SHS may contribute to the green and sustainable treatment for surface modification of natural fiber.

## 1. Introduction

Oil palm mesocarp fiber (OPMF), also known as palm pressed fiber (PPF) is the biomass residue obtained after pressing the palm fruits for palm oil extraction. About 11% of OPMF is generated from the palm fruits after the oil extraction [1]. Generally, OPMF consists of fruit fiber, crushed kernels and shells. Currently, OPMF is mainly utilized as fuel for steam boilers at the mills [2,3]. Being a lignocellulosic material, OPMF has grabbed attention of researchers due to its potential utilization for biocomposite production [4,5], whereby the fiber can be used to reinforce polymer materials such as thermoplastics. Natural fiber-reinforced composites have numerous advantages such as light weight, low-cost, high toughness, and having reasonable strength and stiffness [6,7].

The polar nature of lignocellulose due to the presence of hydroxyl and carboxyl groups in cellulose and hemicellulose causes it to be incompatible with non-polar thermoplastics. The incompatibility leads the biocomposites to form aggregates during extrusion, be difficult to homogenize, and display poor adhesion and limited stress transfer, and show fiber pull-outs in fracture surfaces. Various treatment methods are available in order to increase the interaction between natural fibers and thermoplastics such as the use of coupling agent [8] compatibilizer, crosslinking, hydrothermal treatment [9,10], heat treatment [11], acetylation [12], sodium hydroxide treatment [13], surface treatment [5], chemical grafting, and *etc*. Coupling agents, compatibilizers, crosslinkers and other chemical treatments have been widely used and proven to increase the interaction between natural fiber and polymer matrix [6]. The methods used involve modification of fiber surface either make it more hydrophobic or develop a new linkage between fiber and polymer matrix, which makes the fiber and polymer to have better compatibility and furthermore, better mechanical properties.

Recently, another approach has been introduced to modify the fiber surface that is via hydrothermal treatment [9,10,14,15]. Hydrothermal treatment was found to be able to remove hemicellulose, which is the most hydrophilic and most thermally unstable component in wood, since it has the lowest thermal resistance. Removal of hemicellulose makes the fiber less hydrophilic and this will potentially increase the compatibility of treated wood and polymers and improves the mechanical properties and water resistance of composites [10]. Apart from that, hemicellulose also has a decomposition temperature in the same range of the melt molding temperatures used for common thermoplastics. Degradation of hemicellulose generates volatile substances such as acetic acid and formic acid, hence, if the fiber is blended with a thermoplastic, the hemicellulose component is decomposed and generates unpleasant odor in the working environment.

Looking at the potential of hydrothermal treatment as a method for increasing surface compatibility between fiber and thermoplastics, it is no doubt that steam treatment may have the same capability. To date, the use of steam treatment or steam explosion for lignocellulose has been mainly focusing on the pre-treatment to gain better substrates for enzymatic hydrolysis, which is useful for biofuel production [16]. These studies showed that the use of high pressure steam treatment able to remove hemicellulose. However, the steam treatment used by the researchers involved the use of elevated pressure, which may impose high energy consumption apart from safety issue. 

Superheated steam (SHS) can be an alternative treatment method for lignocellulose. SHS treatment is advantageous compared to steam explosion as it is conducted at atmospheric pressure. To date, SHS has been mainly used for drying [17,18]. Recently, there was report on the use of SHS for treating palm biomass in order to ease the enzymatic hydrolysis of the lignocellulose for sugars production [19,20]. This study was done to reveal the potential of SHS as a novel and alternative treatment method for modification of lignocellulose towards biocomposite production. The treated OPMF obtained in this study was analyzed for its chemical component, thermal stability, chemical structure and morphological characteristic.

## 2. Results and Discussion

### 2.1. Characteristics of Untreated OPMF

Chemical composition analysis showed that untreated OPMF contained cellulose, hemicellulose, and lignin at 42, 32, and 22 wt%, respectively (Table 1). The cellulose, hemicellulose and lignin compositions of OPMF found in this study were similar to those reported elsewhere [1]. 

**Table 1:** Chemical composition of untreated and SHS-treated OPMF.

Sample	Treatment		Chemical composition * (%)			
	Temp (±2 °C)	Time (h)	Hemicellulose	Cellulose	Lignin	Ash
1	0	0	33.10 ± 2.01	42.81 ± 0.69	20.49 ± 3.44	3.59 ± 0.74
2	190	1	26.13 ± 0.18	41.39 ± 0.06	28.44 ± 1.27	4.04 ± 1.02
3		2	22.47 ± 1.67	40.46 ± 2.52	33.38 ± 1.34	3.69 ± 0.49
4		3	19.71 ± 0.72	37.50 ± 0.18	38.72 ± 1.04	4.07 ± 0.14
5	210	1	16.80 ± 2.15	33.75 ± 2.28	45.19 ± 4.66	4.26 ± 0.85
6		2	12.82 ± 1.75	32.85 ± 0.12	49.81 ± 1.91	4.52 ± 0.83
7		3	11.50 ± 1.19	30.75 ± 1.09	52.30 ± 0.92	5.44 ± 0.37
8	230	1	11.42 ± 0.21	33.61 ± 1.10	49.73 ± 0.70	5.24 ± 0.45
9		2	9.42 ± 1.16	34.35 ± 1.33	50.63 ± 1.76	5.59 ± 0.05
10		3	9.71 ± 0.88	28.89 ± 1.75	55.22 ± 1.87	6.18 ± 0.75

* Data provided is mean of duplicate samples.

**Figure 1:**
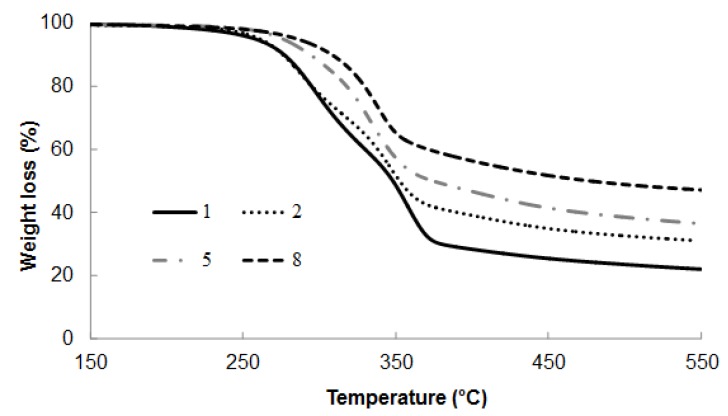
TG analysis for untreated OPMF (1) and SHS-treated OPMF; at 190 °C (2), 210 °C (5) and 230 °C (8), for 1 h of treatment.

Thermal stability of untreated OPMF was evaluated by TGA. Figure 1 shows TG/DTG curves of untreated OPMF (sample 1). It was observed that weight loss occurred at four different regions: (i) 160–220 °C, (ii) 220–320 °C, (iii) 320–390 °C, and (iv) 390–550 °C. The multi-step degradation indicates sequential degradation of multiple components. Based on the chemical analysis, OPMF consists of three main components, *i.e.*, cellulose, hemicellulose and lignin. Lignin, hemicellulose, and cellulose degraded within temperature ranges of 160–900, 220–315 and 315–400 °C, respectively, when heated in TGA at 25–900 °C under purified nitrogen at a flowrate of 120 mL/min and heating rate of 10 °C/min [21]. On the other hand, lignin decomposed at a slower rate compared to the other components in lignocellulose, resulted in degradation over a broader temperature range from 200 to 500 °C [22]. Therefore, it can be concluded that weight loss occurred at 160–220 °C and 390–550 °C was mainly contributed by the degradation of lignin component of OPMF.

**Figure 2:**
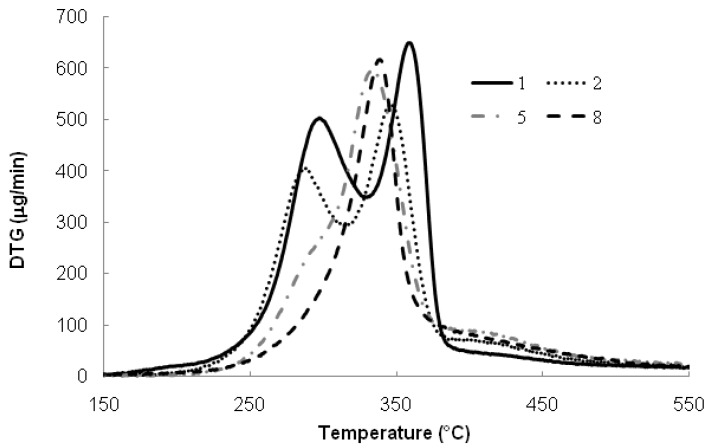
DTG analysis for untreated OPMF (1) and SHS-treated OPMF; at 190 °C (2), 210 °C (5) and 230 °C (8), for 1 h of treatment.

This can be seen clearly in Figure 2, in which the DTG curve suggests a hidden wide peak from the gently sloping baseline. The broader degradation temperature range of lignin can be explained by the presence of various oxygen-containing functional groups in its structure [22]. These complex structures of lignin show various thermal degradation behaviors, since the scissions occurring at different temperatures. Therefore, it is suggested that the temperature range of lignin degradation overlaps with those of hemicellulose and cellulose degradation. 

The degradation occurred within second temperature region ranging from 220 to 320 °C was related to the degradation of hemicellulose and cellulose of the fiber. This is in agreement with a report by Sinha and Rout [23], whereby the glycosidic linkages of cellulose in raw jute fiber started to disrupt at 290 °C. The third stage of weight loss, which occurred at temperature range between 320 and 390 °C, indicates the degradation of cellulose and other non cellulosic materials from the fiber [24]. The intense peaks at 290 and 350 °C in DTG curves (Figure 2) are attributed to the hemicellulose and cellulose decomposition, respectively. 

### 2.2. Effects of SHS Treatment on Chemical Composition of OPMF

OPMF was treated with SHS at various temperatures and treatment times. Table 1 shows the major components of untreated and treated OPMF. Generally, all treated OPMF had higher composition of lignin and lower composition of hemicellulose compared to the untreated OPMF. These results indicate that SHS operated at 190–230 °C for 1 to 3 h under atmospheric pressure selectively removed hemicellulose component, thereby increasing the composition of lignin. Lignin seems to be least affected by the SHS where it can be observed that percentage of lignin was increased as treatment temperature and time increased. Result found in this study was concurrent with a study done by Bahrin *et al.* [20], showed that percentage of lignin in SHS-treated oil palm empty fruit bunch (OPEFB) was increased after SHS treatment. This can be explained by the complex structure of lignin which makes it difficult to be degraded. This is in contrary with hemicellulose whereby the branched structure of hemicellulose makes it easily affected by the SHS treatment. Moreover, the structure of hemicellulose is most unstable because of its amorphous structure [10].

Lignin contains polar hydroxyl groups and non-polar hydrocarbon and benzene rings which make it less hydrophilic [8]. On the other side hemicellulose is hydrophilic due to presence of abundant hydroxyl groups. Therefore, by destruction of hydroxyl in hemicellulose, the percentage of lignin will be higher, thus reduced the overall hydrophilicity of the fiber [9]. In addition, treated samples will be allow the interaction between the non-polar hydrocarbon chains and benzene rings of lignin with hydrophobic polymers. Besides that, hydroxyl groups of lignin will interact with the structure of the fiber [8]. This may promote the adhesion of SHS-treated OPMF to polymer and make it more compatible with the polymer matrix.

Detailed analysis on the chemical composition of the SHS-treated OPMF suggested that the composition of the treated fiber was affected by the treatment temperature and time. It was found that the removal of hemicellulose by SHS treatment was more pronounced at temperatures above 210 °C whereby more than 50% of the hemicellulose was removed compared to the use of lower temperature when treated for 1 h. Moreover, it was also found that prolonged treatment time of up to 3 h may reduce the hemicellulose content to nearly half of the original value, even at lower temperature, *i.e.*, 190 °C. This indicates that hydrolysis of hemicellulose by SHS was affected by both temperature and retention time. However, the use of high temperature may hydrolyze the cellulose component of OPMF. This was proven when percentage of cellulose started to decrease at temperature 210 °C and higher. At temperature a 230 °C for 3 h of treatment, the cellulose content was decreased notably. This is in agreement with Ando *et al*. [25] which reported that cellulose started to degrade when the temperature of hot water extraction was over 230 °C. 

Furthermore, prolonged the retention time up to 3 h for SHS treatment at 230 °C did not give a marked difference in hemicellulose content. About 8%–9% of hemicellulose was still left in the treated OPMF, as seen in Table 1. This could be the result of recalcitrant hemicellulose, which is closely linked to lignin through covalent bonds such as ester and ether bonds. This kind of bonding may require more energy to break.

Partial removal of hemicellulose and elimination of low degradation temperature lignin parts, may cause the SHS-treated OPMF to be more ductile [26]. The removal of both hemicellulose and lignin which are considered as internal constraints may lead to a closer packing of the cellulose chain. In other words, the removal of hemicellulose and low degradation temperature of lignin may cause improvement in fiber strength and its mechanical properties. 

**Figure 3:**
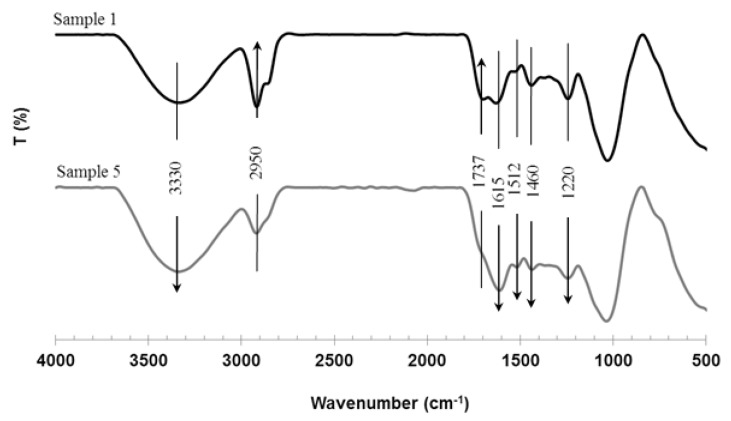
FTIR spectra of the untreated OPMF (1) and SHS-treated OPMF treated at 210 °C for 1 h (5).

Alteration in chemical components of SHS-treated OPMF (sample 5) can also be shown by the changes in its chemical structure and compared with the untreated OPMF (sample 1) as seen in Figure 3. From this FTIR spectrum, it is seen that the dominant absorption peaks were found at 3,330 and 2,950 cm^−1^, which were attributed by the stretching vibrations of –OH groups and the C–H stretching, respectively. Peak stretching around 3,330 cm^−1^ was increased after the extraction [10]. This peak was known to be highly influence by moisture, hence it is difficult to discuss about changes of hydroxyl groups from this band. However, the intensity of the band around 2,950 cm^−1^ decreased after the steam treatment which derived from C–H groups after fiber treatment might be attributed to the removal of hemicelluloses. 

Meanwhile a small shoulder peak at 1,737 cm^−1^ is assigned to the characteristic carbonyl groups in hemicellulose and/or lignin. This band was decreased after the treatment which could be due to the removal of hemicellulose. During steam treatment, deacetylation occurred, whereby hemicellulose will be degraded to form acetic acid [14]. 

The peaks at 1,512 and 1,460 cm^−1^ are indicative of the aromatic C=C stretch of aromatic vibrations in bound lignin [27]. The intensity of these peaks increases in the treated OPMF and become more apparent when the treatment time exceeds the temperature of 210 °C for 1 h, reflecting a higher fraction of lignin in the treated samples. The peaks in the 1,500–1,000 cm^−1^ region changed as SHS was being applied to the OPMF. It is supposed that the absorbance peaks at 1,362 and 1,220 cm^−1^ originate from C–H ester bands and C–O stretching vibrations are due to partial acetylation of hydroxyl groups in both polysaccharides and residual lignin [28].

The increment of the respective peaks revealed a higher percentage of lignin after SHS treatment which corroborates the earlier statement. The intensity of the band related to C-O-C pyranose ring skeletal vibration and C-O anti-symmetric stretching, around 1,100 cm^−1^, increased after the treatment, which also shows the higher lignin ratio in treated samples [21]. The 900/1,000 cm^−1^ peak represents the glycosidic C–H deformation with ring vibration contribution and OH bending in the treated OPMF, which indicates the typical structure of cellulose [29], has not changed. From the FTIR result, it was suggested that during the SHS treatment, reactions occurred randomly on the entire OPMF surface.

### 2.3. Effects of SHS Treatment on Thermal Stability of OPMF

Studies of the thermal properties of OPMF are very important in order to estimate the fiber’s potential application in reinforced-polymer composite processing because the processing temperature for many polymeric materials exceeds 200 °C. Figure 1 and Figure 2 show the TG and DTG curves of untreated and SHS-treated OPMF. Based on both figures, it is clearly seen that the degradation temperature greatly increased after the SHS treatment. 

The multi-step degradation of untreated OPMF (sample 1) gradually changed to single step degradation for treated OPMF as seen in the TG curves, indicating that the component in the treated OPMF gradually changed with the SHS treatment. This can be clearly seen from the DTG curves (Figure 2). The peak at around 300 °C which represents hemicellulose decomposition was rapidly decreased and nearly disappeared in the SHS-treated samples at high temperature, *i.e.*, 230 °C. In other words, the unstable hemicellulose ingredient was removed during the SHS treatment, resulting in an increase in the degradation start point [10]. However as discussed earlier, at high temperature cellulose will also partly degraded, therefore, the disappearance of a broad peak at 160–340 °C for untreated samples can be assumed as a thermal degradation of mainly hemicellulose and cellulose. SHS treatment operated at higher temperature and prolonged time may also affect the degree of polymerization of cellulose, which can explain the lower DTG peaks of OPMF after the treatment.

The residue remained after heating the fiber up to 550 °C for both untreated and SHS-treated OPMF indicates the presence of carbonaceous materials in the OPMF. The distinct observation between the amount of the residue for untreated and SHS-treated OPMF is attributed by the lignin content and inorganic compounds (ash) which is in parallel with the chemical composition of OPMF presented in Table 1. As being mentioned earlier, lignin has a wide degradation temperature (160–900 °C), and lignin is less prone to degradation due to its complex structure. This explains why lignin is less affected during steam treatment and hence, contributed to the large amount of residue after TG analysis of treated OPMF.

Increased in thermal stability of treated OPMF is shown in detail in Table 2. The table shows the decomposition temperature of fiber at 5, 20 and 50% weight loss (*T*_5%_*, T*_20%_, and *T*_50%_), the onset temperature of TGA and peak temperature of DTG. The *T*_5%_ of untreated OPMF was recorded at 258 °C. The *T*_5%_ value was increased to 261, 276 and 285 °C, when the OPMF was treated for 1 h at 190, 210 and 230 °C, respectively. This observation suggested that thermal stability of the OPMF was indeed visibly improved after the SHS treatment. 

**Table 2:** Degradation temperature at 5, 20 and 50% fiber degradation, obtained by TGA of untreated and SHS-treated OPMF.

Sample	Temp (°C)	Time (h)	*T*_5%_ (°C)	*T*_20%_ (°C)	*T*_50%_ (°C)	*T*_p_ (°C)	Residue at 550 °C (%)
1	0	0	257.7	294.8	348.1	299.6,	22.01
						359.9	
2	190	1	260.9	295.1	352.5	287.7,	31.11
						346.6	
5	210	1	276.0	317.9	374.3	334.1	36.63
8	230	1	285.3	329.5	476.9	339.2	47.14

*T_p__%_* represents the onset decomposition temperature of 5, 20 and 50% weight loss, *T_p_* represents peak temperature of DTG.

Overall, these results suggested that the high temperature of thermal decomposition and high residual mass of the OPMF obtained after SHS treatments is attributable to the selective removal of hemicellulose. These results are consistent with results obtained from the FTIR and chemical analysis as discussed in the earlier section.

### 2.4. Effect of SHS Treatment on the Morphology of OPMF

**Figure 4:**
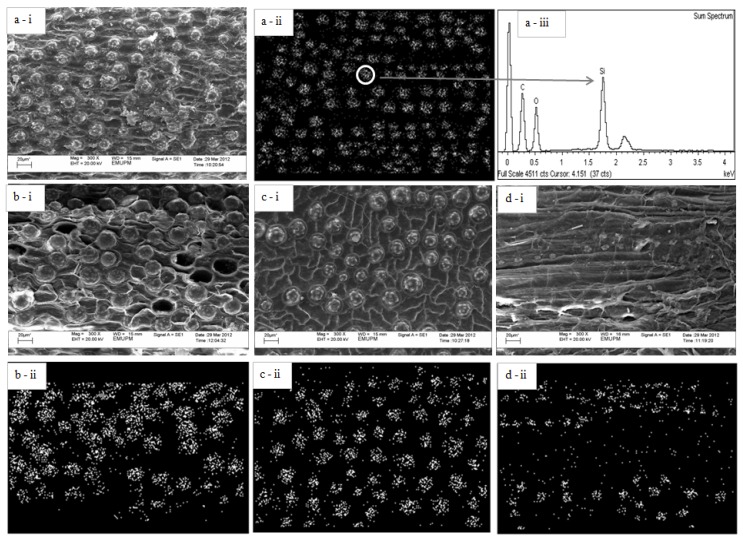
SEM images of untreated OPMF (a-i), SEM mapping of untreated OPMF (a-ii), microanalysis spectrum of untreated OPMF indicate the particle is silica (a-iii), SHS-treated OPMF at 190 °C (b-i & ii), 210 °C (c-i & ii) and 230 °C (d-i & ii) for 1 h, show reduction of silica bodies after SHS treatment.

Apart from modifying the chemical composition and thermal stability of the OPMF, SHS treatment also resulted in morphological changes as well as chemical changes on the OPMF surface. Figure 4a-i shows the surface morphology of untreated OPMF, whereby silica bodies were observed to deposit on the entire fiber surface. 

Another study shows that silica bodies were observed to be present on the entire surface of OPMF strands [30]. The SEM image is also similar with that of OPEFB, where silica bodies are found to be spread at the entire fiber surface [31]. Figure 4b-i,c-i,d-i show the morphology of OPMF after SHS treatment at 190 °C, 210 °C and 230 °C for 1 h. Based on these figures, it was found that SHS treatment was able to remove silica bodies on the OPMF surface.

In Figure 4(a-ii) shows the image of SEM mapping, where the silver particles are represents the silica bodies. The finding is supported by EDX analysis, which is shown in Figure 4(a-iii). The spectrum of the elements present in the fiber surface demonstrated that the particles were silica. 

In Figure 4b-ii,c-ii,d-ii which are the SEM mapping of the micrographs, it can be clearly seen that the amount of silica bodies was decreased after the SHS treatment. The silica content in the untreated and SHS-treated OPMF is shown in Table 3. From the analysis, the silica content was reduced after the steam treatment, hence the result obtained was in agreement with the SEM micrograph which show the silica bodies were decreased after the SHS treatment. 

**Table 3:** Content of silica in untreated OPMF and after superheated steam treatment.

Sample	Treatment		Silica (ppm)
	Temp (±2 °C)	Time (h)	
1	0	0	224.8
6	210	1	203.9
9	230	2	198.3
10	230	3	93.4

This observation is similar to those reported by others studies [18,20], whereby SHS treatment may remove silica bodies from OPEFB. On the other hand, SHS treatment was also found beneficial to loosen up the structure of the OPMF, as seen in the Figure 4d-i. The loose structure of treated fiber can be explained by the removal of hemicellulose which generally acts as crosslinker between cellulose microfibrils and fills the gap between cellulose and lignin. Therefore, when hemicellulose is removed, the tightly packed cellulose microfibrils tend to become loose. Loosening of OPEFB structure was observed after thermal pretreatment at 240 °C [32].

The surface of SHS-treated samples also became rougher as can be seen in Figure 4b–i–d–i, compared to the untreated OPMF, which is attributed by the removal of silica bodies. Generally, from the observation of SEM micrograph in Figure 4a,d, the average diameter of massive silica bodies is about 10–20 μm for the untreated OPMF. The rougher surface of SHS-treated OPMF is an advantage as the scar-like surface of fiber obtained after dislodging of silica bodies will enhance the anchor effect to the polymer matrix, which will subsequently improve the interaction between the two materials [31]. Moreover, the uneven surface of fiber is more advantageous to help the diffusion and penetration of melt polymer chains into the fiber [31].

### 2.5. Wide-Angle X-ray Diffraction (WAXD) Analysis

**Figure 5:**
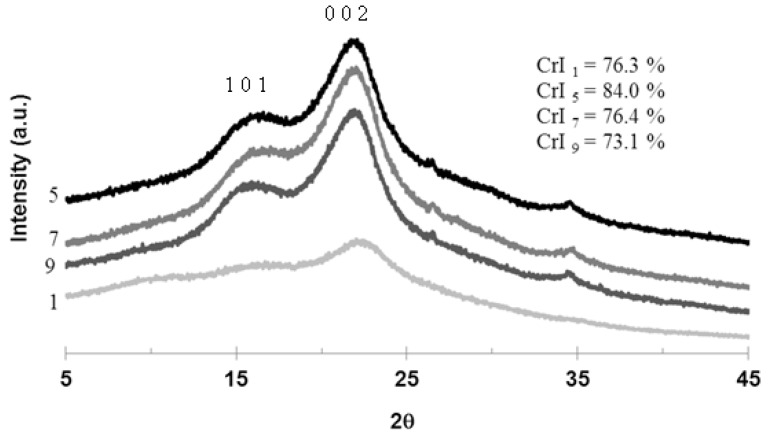
X-ray diffraction patterns of untreated OPMF (1), SHS-treated OPMF; at 210 °C for 1 h (5), 210 °C for 3 h (7) and 230 °C for 2 h (9) of treatment.

The WAXD patterns of untreated and SHS-treated OPMF in Figure 5 show the natural occurring cellulose form known as cellulose I [33]. It was reported that the peak at about 2θ = 23 is the peak for the crystalline portion of biomass (*i.e.*, cellulose) and the peak at about 2θ = 18 corresponds to the amorphous region [34,35]. It can be seen that the diffraction peak at 23° (0 0 2) is wide and broad for untreated OPMF, however, the peak was sharper and narrower in SHS-treated OPMF, hence indicating a higher degree of crystallinity in the treated fibers. The crystallinity index for untreated OPMF was 76.3% and this increased to 84.0% after treatment at a temperature of 210 °C for 1 h. However after prolonged treatment *i.e.*, 3 h, the sample shows a decrease in crystallinity index (to 76.4%). This might be due to the long treatment that disrupts the structure of cellulose. The crystallinity index of OPMF decreased to 73.1% at a higher temperature, 230 °C after 2 h. The increased of crystallinity in treated samples can be attributed to the removal of cementing amorphous component, which was hemicellulose as shown in Section 3.2. Fiber with high crystallinity might improve the mechanical properties of the composite. It will promote the resistance to cracks which may contribute to better mechanical properties. After the treatment, the hydrogen bonds between cellulose molecules resulted in an ordered system. Individual fibrillar units consist of long periods of ordered regions interrupted by completely disordered regions. 

## 3. Experimental

### 3.1. Materials

In this study, oil palm mesocarp fiber (OPMF) was obtained from FELDA Serting Hilir Palm Oil Mill, Negeri Sembilan in Malaysia. At first, OPMF with average length of 20–30 mm was physically treated to disintegration, followed by washing, cleaning, sorting and sun dried. Dried OPMF was then stored in sealed plastic bag at room temperature (±24 °C) prior to use. Sulfuric acid (90% purity) and potassium hydroxide were supplied by Merck, Darmstadt, Germany and sodium chlorite was supplied by Acros Organics, Geel, Belgium.

### 3.2. Superheated Steam Treatment

The superheated steam machine consists of a two separate parts which are a stainless steel heating chamber and a heater kind of boiler. The boiler operated at power of 6.3 kW and an evaporation rate of 9.45 kg/h. Steam was produced from the boiler and injected to the samples placed in the heating chamber. An electric heater, rated at 8 kW, was installed in the heating chamber to maintain the steam temperature under superheated conditions. Superheated steam treatment was carried out in batch experiments. The OPMF was treated with the superheated steam oven (QF-5200C, Naomoto Corporation, Osaka, Japan) at 190, 210, and 230 °C (±2 °C) for 1 to 3 h, respectively under ambient pressure. The SHS machine was preheated until the desired temperature was achieved and stable. About, 5 g of OPMF were placed on stainless steel mesh under the designated conditions. Samples were immediately removed from the heating chamber at the end of treatment. Moisture content of OPMF was measured before and after SHS treatment. Treated samples were then kept in a desiccator prior to characterization.

### 3.3. Characterization and Analysis of Untreated and Treated OPMF

#### 3.3.1. Chemical Composition Analysis

The chemical composition of the untreated and treated OPMF was determined according to the procedure described by Iwamoto *et al.* [36]. For lignin removal, the OPMF was soaked in 5 wt% sodium chlorite (NaClO_2_) solution adjusted with sulfuric acid to a pH of 4 to 5. The fibers were soaked at 70 °C for 1 h followed by washing with deionized water until the pH became neutral. Hemicellulose was extracted by bleaching the fibers with 6 wt% potassium hydroxide (KOH) solution at 25 °C for 24 h. The sample was then rinsed with deionized water until the pH 7. Ash was determined according to Varley [37] with some modification. Samples were heated in the furnace at temperature 550 °C for 2 h. The crucibles were cooled in a desiccator and weigh it. The crucible and OPMF was dried at 105 °C overnight prior analyses. Heavy metal *i.e.*, silica was determined by using Inductively Coupled Plasma (ICP) analysis. The samples were digested according to ashing and preparation of ash solution, the standard methods as described in MS 677: Pt. I–VIII: Part II [38].

#### 3.3.2. Analytical Measurements

Thermogravimetric analysis (TGA) was conducted on a TG analyzer model EXSTAR6000 TG/DTA6200 (Hitachi High-Tech Science Corporation, Tokyo, Japan) in order to confirm the change in the composition of the treated OPMF. The OPMF powder sample (6–8 mg) was placed on an aluminum pan. The sample was heated from 50–550 °C at a heating rate of 10 °C/min under nitrogen flow of 100 mL/min. The corresponding weight loss (μg) and its derivative DTG (μg/min) were recorded.

Functional groups of OPMF were examined using a Perkin Elmer Spectrum GX Fourier transform infra red (FT-IR) spectrometer (Perkin Elmer, Waltham, MA, USA) via the attenuated total reflectance (ATR) method. Prior to analysis, samples were oven-dried for 24 h. The spectrum was recorded over the wavenumber ranging between 400 and 4,000 cm^−1^. The spectra were the average of 16 scans at a spectral resolution of 4 cm^−1^. 

The surface morphology including microanalysis of OPMF and steam-treated OPMF samples was observed under a scanning electron microscopy (SEM) with a model LEO 1455 VPSEM (LEO Electron Microscopy Ltd., Cambridge, UK) with an Oxford Inca EDX (Oxford Instruments, Oxfordshire, UK). For SEM/EDX analysis, oven-dried OPMF samples were mounted on the stub and gold-coated for 180 sec prior to SEM/EDX observation. The SEM/EDX micrographs were obtained with an acceleration voltage of 20 kV.

The crystallinity of the untreated and treated OPMF were measured using Wide-angle X-ray diffraction (WAXD) (Rigaku Corporation, Tokyo, Japan) with Cu Kα radiation source (λ = 0.154 nm) at 40 kV and 30 mA. Samples were scanned at 2θ from 5 to 50° at room temperature. The crystallinity index (CrI) was calculated from XRD data and determined based on the formula by Segal *et al*. [35]:

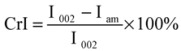
(1)
which I_002_ is the intensity for the crystalline portion of sample, *i.e.*, cellulose at about 2θ = 22.5 and I_am_ is the peak for the amorphous portion, *i.e.*, cellulose, hemicellulose and lignin, at about 2θ = 18.7. In this study, it should be noted that peak of I_002_ was at about 2θ = 23 and second highest peak which is I_am_ was at about 2θ = 18.

## 4. Conclusions

In the present work, it is shown that SHS treatment is useful in the modification of lignocellulose fiber for biocomposite production. Chemical composition, thermal stability and morphological properties of the OPMF were greatly affected by SHS treatment. Chemical composition and FTIR analysis of the SHS-treated OPMF revealed the highly selective removal of hemicellulose, indicating the success of SHS treatment as a treatment method for preparing less hydrophilic lignocellulose fiber for biocomposite production. Moreover, SHS-treated OPMF also exhibited improved thermal stability properties, whereby the thermal degradation temperature increased from 258 °C for untreated OPMF to 285 °C for SHS-treated OPMF, thus making the fiber a promising candidate in polymer composite processing. Morphological analysis revealed that the SHS-treated OPMF has a rougher surface due to the removal of silica bodies. Overall, it can be concluded that SHS treatment has great potential application in reinforced-polymer composite processing as it may provide lignocellulose fiber with better physical, chemical and thermal properties which in turn may produce biocomposites with enhanced characteristics. Moreover, the non-chemical and environmentally friendly characteristics of this treatment give extra value to this novel and green method.
